# Starting Home Telemonitoring and Oxygen Therapy Directly after Emergency Department Assessment Appears to Be Safe in COVID-19 Patients

**DOI:** 10.3390/jcm11237236

**Published:** 2022-12-06

**Authors:** Rosaline van den Berg, Celisa Meccanici, Netty de Graaf, Eric van Thiel, Suzanne Schol-Gelok

**Affiliations:** 1 Science Office, Albert Schweitzer Hospital, Albert Schweitzerplaats 25, 3318 AT Dordrecht, The Netherlands; 2 Emergency Department, Albert Schweitzer Hospital, Albert Schweitzerplaats 25, 3318 AT Dordrecht, The Netherlands; 3 Department of Pulmonology, Albert Schweitzer Hospital, Albert Schweitzerplaats 25, 3318 AT Dordrecht, The Netherlands

**Keywords:** COVID-19, home telemonitoring, oxygen therapy, hospital discharge

## Abstract

Background: Since data on the safety and effectiveness of home telemonitoring and oxygen therapy started directly after Emergency Department (ED) assessment in COVID-19 patients are sparse but could have many advantages, we evaluated these parameters in this study. Methods: All COVID-19 patients ≥18 years eligible for receiving home telemonitoring (November 2020-February 2022, Albert Schweitzer hospital, the Netherlands) were included: patients started directly after ED assessment (ED group) or after hospital admission (admission group). Safety (number of ED reassessments and hospital readmissions) and effectiveness (number of phone calls, duration of oxygen usage and home telemonitoring) were described in both groups. Results: 278 patients were included (*n* = 65 ED group, *n* = 213 admission group). ED group: 23.8% (*n* = 15) was reassessed, 15.9% (*n* = 10) was admitted and 7.7% (*n* = 5) ICU admitted. Admission group: 15.8% (*n* = 37) was reassessed, 6.5% (*n* = 14) was readmitted and 2.4% (*n* = 5) ICU (re)admitted. Ten patients died, of whom 7 due to COVID-19 (1 in ED group; 6 in the admission group). ED group: median duration of oxygen therapy was 9 (IQR 7–13) days; the total duration of home telemonitoring was 14 (IQR 9–18) days. Admission group: duration of oxygen therapy was 10 (IQR 6–16) days; total duration of home telemonitoring was 14 (IQR 10–20) days. Conclusion: it appears to be safe to start home telemonitoring and oxygen therapy directly after ED assessment.

## 1. Introduction

The COVID-19 pandemic led to severe stress on hospital capacity worldwide. Although most COVID-19 cases can be managed in the outpatient setting, approximately 10–15% of the patients require hospitalization due to the need for oxygen supply during the 2020–2021 pandemic [1,2,3].

In order to reduce the length of hospital stay, home telemonitoring including oxygen supply therapy emerged during the COVID-19 pandemic as a promising and powerful modality to treat and monitor COVID-19 patients at home [2,4,5,6].

Home telemonitoring is now implemented in several hospitals as a standard of care and is considered a safe strategy for monitoring COVID-19 patients after hospital discharge [7,8,9,10]. Published studies on the safety of home telemonitoring started directly after an assessment at the Emergency Department (ED) [11,12,13,14] or on the safety of home telemonitoring and oxygen therapy [15,16] are sparse. As far as we know, there are only four studies describing the results of home telemonitoring and oxygen therapy started directly after ED assessment; one was performed in Guadeloupe [17] and three in the United States of America [18,19,20].

In November 2020, the Albert Schweitzer hospital (ASz)–a large teaching hospital in Dordrecht, The Netherlands-developed a care pathway, enabling to send a specific group of confirmed COVID-19 patients home with home telemonitoring and oxygen therapy directly after assessment at the emergency department (ED) [1,2]. Hence, fewer COVID-19 patients needed hospital admission and hospital capacity could be used more efficiently.

In this study, we aim to show that home telemonitoring and oxygen therapy of COVID-19 patients directly started after ED assessment is safe and effective. Moreover, we investigated the experience and satisfaction of all COVID-19 patients that received home telemonitoring.

## 2. Materials and Methods

### 2.1. Patients

All patients ≥18 years old with a polymerase chain reaction (PCR)-confirmed COVID-19 infection, and receiving home telemonitoring provided by the ASz hospital in the period November 2020 to February 2022, were included in this study.

**Figure 1:**
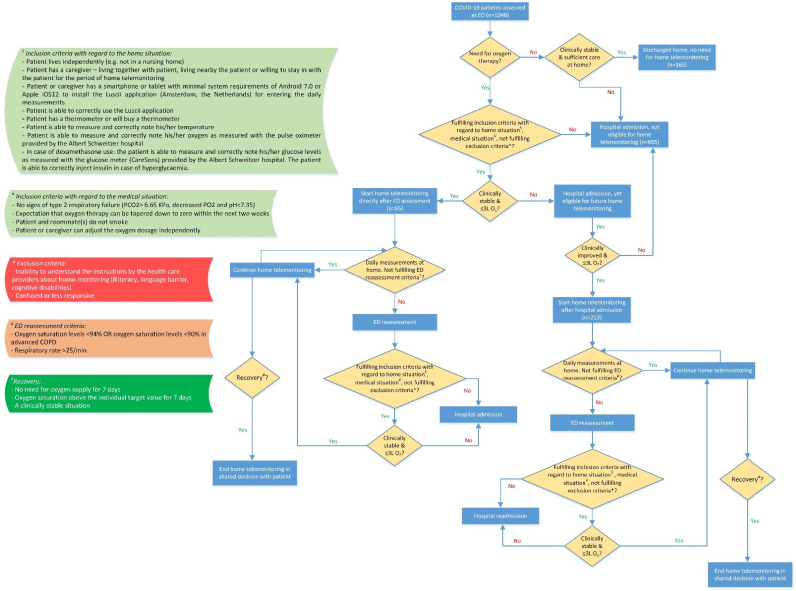
Flowchart of in- and exclusion criteria and decisions for home telemonitoring.

Home telemonitoring was only started when patients needed oxygen therapy and should therefore be monitored more closely. If patients did not need oxygen therapy, no home telemonitoring was started. Additionally, if patients did not fulfill the criteria with regard to the home situation (amongst others, patients had to live independently (e.g., not in a nursing home), had to have a caregiver living with or nearby the patient or willing to stay in with the patient during home telemonitoring, and had to have a smartphone or tablet to install the Luscii application), or did not fulfill the criteria with regard to the medical situation (e.g., the patient smokes), or if they fulfilled the exclusion criteria (e.g., unable to understand the instructions by the health care providers about home telemonitoring), patients could not receive home telemonitoring (Figure 1). Then, it depended on the medical situation (clinically stable or not) and the amount of needed oxygen if they could be sent home with home telemonitoring directly after ED assessment or if they were admitted. Therefore, two different home telemonitoring groups could be defined. The first group consisted of COVID-19 patients who started with home telemonitoring directly after ED assessment (ED group). This group was clinically stable at ED assessment and received ≤3 L oxygen therapy. The second group consisted of COVID-19 patients who started with home telemonitoring after hospital admission in the ASz (admission group) when they were clinically stable and needed ≤3 L oxygen therapy (Figure 1).

In the ASz hospital, all patients are informed upon admission or ED assessment, that their coded data might be used for research purposes. In case of disapproval, this objection can be expressed to the treating physician, nurse, desk employee, or data protection officer, and the objection is noted in the Electronic Health Record (EHR).

During the COVID pandemic, it was not feasible to obtain signed informed consent. Signing informed consent would have introduced additional risks (e.g., additional contacts with the patient) which are in conflict with the guidelines of the National Institute for Public Health and the Environment (RIVM) [14,21]. Moreover, some patients were not able to sign informed consent, due to serious respiratory illness. The pivotal importance to gain knowledge about COVID-19 was surpassing the rights of an individual patient to be fully informed with written information and to sign informed consent, as in normal circumstances.

Therefore, we deemed it ethically acceptable in these exceptional times to accept only a verification of the general objection in the EHR for retrospective studies, and it was judged by the local institutional review board that no additional informed consent was necessary. Only data from patients without objection were used.

### 2.2. Home Telemonitoring

If the patient met the inclusion criteria for home telemonitoring, a pulmonary nurse practitioner provided further instructions. The Luscii app (Luscii Healthtech BV, Utrecht, The Netherlands) was installed on the patient’s smartphone or tablet. Devices were provided to monitor vital parameters at home. Vital parameters consisted of i.a. oxygen saturation measured by pulse oximeter and respiratory rate. Vital parameters were measured four times a day and completed into the Luscii app by the patients themselves (eight measurements/day). Individual thresholds were set for each patient depending on their medical history. Above/below those thresholds, an alert triggered action of the home telemonitoring team, consisting of pulmonary nurse practitioners and medical doctors supervised by pulmonologists. They monitored all patient information sent by the Luscii app on the Luscii dashboard. This dashboard was updated several times a minute. The attending pulmonologist decided whether the patient needed to be reassessed at the ED. When arrived at the ED, patients were seen by a physician and a complete assessment was performed including measurements of vital parameters. When there was no need for hospitalization, patients were discharged and continued in the home telemonitoring program. Other patients were admitted after ED reassessment. The decision to admit a patient to the ICU was based on the need for intubation, and on the chances of recovery from ICU admission. This decision was always made in a shared decision by the attending intensivist with the patient and relatives.

#### 2.2.1. Continuation of Home Telemonitoring

The home telemonitoring team actively contacted patients by phone to control clinical follow-up once daily as long as an oxygen supply was needed. Patients were also instructed to contact the home telemonitoring team in case of deviating measurements or progressive complaints. Additionally, in this situation, the attending pulmonologist decided whether the patient needed to be reassessed at the ED.

#### 2.2.2. Ending Home Telemonitoring

Home telemonitoring ended in a shared decision with the patient in case of recovery or directly in case of (re-)admission or death. Recovery was defined as no need for oxygen supply for 7 days, oxygen saturation above the individual target value for 7 days and a clinically stable situation.

Directly after ending home telemonitoring, patients received a digital questionnaire about their experience and satisfaction with home telemonitoring. This questionnaire was developed by the Quality, Safety and Innovations department of the ASz to evaluate the provided care, and not specifically for research purposes of this study.

### 2.3. Data Collection

For this study, data on among others gender, age, body mass index (BMI), comorbidities, COVID Outcome Prediction in the Emergency Department (COPE) score [22], medication, (re)admission, hospital stay and oxygen usage were extracted retrospectively from the EHRs. Data including measurements of vital parameters, duration of home telemonitoring and number of telephone contacts with the home telemonitoring team were extracted from the Luscii app.

### 2.4. Statistical Analysis

We provided insight into our primary outcome safety by descriptive statistics, expressed as the number of reassessments at the ED, number of readmissions, number of alerts, compliance and in- and out-of-hospital mortality in the ED group. Compliance is defined as the performance of measurements 4 times a day—regardless of which measurements it was (either respiratory rate or oxygen saturation or both). To describe our secondary outcome effectiveness, expressed as the number of phone calls, duration of home telemonitoring and oxygen usage we also used descriptive statistics.

To put these results in perspective, we presented primary and secondary outcomes, as well as patient and disease characteristics of the ED group and the admission group side by side.

The results of the questionnaires were anonymous and could not be assigned to specific groups. These data are, therefore, reported for all included patients together using descriptive statistics.

Continuous variables were reported as mean ± standard deviation (SD) or, in case of skewed distribution, as the median and interquartile range (IQR, 25th to 75th percentile). Categorical variables were presented as numbers and proportions.

Statistical analyses were performed in STATA software version 14 (StataCorp, College Station, TX, USA).

## 3. Results

Data were available of *n* = 278 patients that received home telemonitoring in the period November 2020–February 2022; *n* = 65 directly after ED assessment, *n* = 213 after hospital admission. Patient and disease characteristics are described in Table 1. The mean age of the ED group was 57.1 (SD 12.4) and 43.1% was female. The mean BMI was 29.8 (SD 5.8) and the most frequent underlying health conditions were chronic cardiovascular diseases (40%), followed by malignancies (18.5%). The median COPE score for predicting death was 5.4 (IQR 2.7–9.8); the median COPE score for predicting of need for ICU admission was 14.2 (IQR 9.8–19.6). Patient and disease characteristics of the admission group were almost equally distributed (Table 1).

Patients in the ED group were not treated with REGEN-COV or remdesivir, as REGEN-COV and remdesivir were not administered in the ED. One-third of the patients in the ED group were treated with antibiotics, anticoagulants and dexamethasone. In the admission group, eighty to ninety percent of the patients were treated with these medications (Table 1).

Median length of stay of the admission group was 4 days (IQR 2–7); 12 patients (5.6%) were admitted to the ICU.

### 3.1. Safety of Home Telemonitoring

Of the patients in the ED group, 23.8% (*n* = 15) were reassessed at the ED, 15.9% (*n* = 10) was admitted to the hospital and 7.7% (*n* = 5) were admitted to the ICU (Table 1). A median of 3 alerts (IQR 1–6) per patient was reported with regard to low oxygen saturation levels, and a median of 2 alerts (IQR 0–10) for tachypnea.

In the admission group, 15.8% (*n* = 37) was reassessed at the ED, 6.5% (*n* = 14) was readmitted to the hospital and 2.4% (*n* = 5) was (re)admitted to the ICU. The readmission rate for the ED group and admission group combined was 8.7%. Per patient, a median of 1 alert (IQR 0–4) with regard to low oxygen saturation levels, and a median of 1 alert (IQR 0–8) for tachypnea was reported. Compliance was high in both groups throughout the total duration of home telemonitoring: median 100% (IQR 100–100) on day 1, median 100% (IQR 75–100) on day 5, median 100% (IQR 75–100) on day 9 in the ED group and median 100% (IQR 100–100) on day 1, median 100% (IQR 100–100) on day 5, median 100% (IQR 75–100) on day 9 in the admission group. Patients that were recovering/improving were less compliant than the patients that required more oxygen therapy. A total of 15,885 measurements of oxygen saturation levels and tachypnea were registered, of which 5.1% triggered an alert.

In total, 10 patients died, 2 of whom were in the ED group and 8 were in the admission group. In the ED group, 1 patient and in the admission group 6 patients died as a result of COVID-19 infection. The other patients died due to pre-existing malignancies and perforated sigmoid diverticular disease, not related to COVID-19.

### 3.2. Effectiveness of Home Telemonitoring

In the ED group, the median duration of oxygen therapy was 9 (IQR 7–13) days, and the total duration of home telemonitoring was 14 (IQR 9–18) days. During home telemonitoring a median of 9 (IQR 7–12) of the daily scheduled phone calls were performed by the physicians. In total during the complete home telemonitoring period, patients stated a median of 4 (IQR 1–13) times that they would have appreciated an additional consult with their physician, on top of the daily scheduled phone calls.

Numbers for the admission group were similar, except for the wish to have an additional consultation with the physician on top of the daily scheduled phone calls (median 11.5 (IQR 3–24)) (Table 2).

In *n* = 5 patients in both the ED group and the admission group, home telemonitoring was ended within 24 h due to (re)admission.

### 3.3. Experience and Satisfaction

In total, 58 patients responded to the questionnaire about their experience and satisfaction with home telemonitoring. The majority of patients were satisfied with home telemonitoring, regarding the information (71%) and instructions they had received (62–84%). Patients also indicated that health care providers had the warranted expertise (84%), as well as sufficient time and attention for patients and their caregivers (53–62%). Patients felt safe at home and were glad they could recover at home instead of in the hospital (86–94%). Overall, they rated the home telemonitoring with a median score of 9 out of 10 (IQR 8–10) (Table 3).

## 4. Discussion

Home telemonitoring and oxygen therapy directly started after ED assessment appears to be safe in patients with COVID-19 in terms of an acceptable number of reassessments, readmissions, compliance, alerts and mortality rates. Additionally, home telemonitoring seems to be effective in terms of the number of phone calls, duration of home telemonitoring and oxygen usage, especially during a pandemic.

A set of clear benchmarks to measure our results on the safety and effectiveness of home telemonitoring and oxygen therapy directly started after ED assessment could not easily be defined, because the literature on this specific topic is sparse.

In order to make the conclusion that home telemonitoring is safe, it is important to put our results in perspective to the results of other studies. By putting our safety results in perspective to existing literature, we could conclude that our number of ED reassessments and number of readmissions were almost similar to existing literature that concluded that home telemonitoring is safe [17,18,19,20]. This judgment is subjective, however, there is no gold standard to rely on. To our knowledge, there are only four studies describing the safety and efficacy of home telemonitoring and oxygen started directly after ED assessment [17,18,19,20]. Readmission rates reported in these studies range from 4.8% in a group of patients managed by a dedicated medical team on ambulatory care for patients requiring oxygen therapy to 20% in a group of patients requiring oxygen therapy managed by the ED physicians [17], to 9.3% in the study by Kuo [19] and 17.5% in the study by Terp [18]. Our readmission rate of 15.9% lies in that range. In the study by Steel, the readmission rate of the patients receiving home telemonitoring and oxygen therapy are presented combined with patients receiving home telemonitoring without oxygen therapy [20]. With regard to reassessments at the ED, our rate of 23.8% is also in line with reported reassessment rates by others: 6.7% reported by Kuo et al. [19], 21.6% reported by Steel et al. [20] and 23.1% reported by Terp et al. [18]. Viel et al. [17] did not report on ED reassessment rates. The mortality rate (3.1%) in our study is slightly higher than the mortality rates reported by Terp et al. (1.4%) [18] and Steel et al. (1.5%) [20], however, in those studies, it is not clear if this rate is an all-cause mortality rate or COVID-19 specific. The other studies did not report on mortality rates.

Reasonably, home telemonitoring started directly after ED assessment prevented an admission and home telemonitoring started after admission shortened the length of stay. This, in combination with expected duration of oxygen usage and home telemonitoring, and no extensively frequent phone calls, led to our conclusion that home telemonitoring is effective.

As presenting the data of patients in the ED group and the admission group together in one group is not representing the scope of our study, we presented data of the groups side by side. By putting the results of the ED group in perspective to our admission group, it was noticed that the admission rate in the ED group was somewhat higher. We expected that home telemonitoring in the ED group probably started earlier in the disease course than in the admission group as patients directly sent home from the ED for telemonitoring are probably in an earlier phase of COVID-19 infection. Patients with COVID-19 could develop respiratory failure within a few days due to pulmonary invasion of COVID-19 and auto-inflammation. Hence, in our opinion, patients in the ED group have a higher chance to deteriorate respiratory rapidly than patients in the admission group who had shown to be clinically stable for a couple of days. Unfortunately, we could not explore this hypothesis, because we did not have exact data describing the first day of illness caused by the COVID-19 infection in both groups

Patients in the admission group more frequently indicated the wish to have contact with a healthcare provider in addition to the scheduled telephone contacts, compared to patients from the ED group. We suppose that patients who were admitted might have the feeling of ‘knowing’ the healthcare providers, which could lower the threshold to contact healthcare providers. Compliance (completion of measurements in home telemonitoring) could also be improved by lowering the threshold to contact a healthcare provider. Lowering this threshold also in the experience of the ED group might provide the opportunity to intervene earlier.

A limitation of our study is the small sample size, which might have an impact on the results. Yet, the sample size is comparable to the sample size in similar studies [17,18,19]. To be noticed, health care is organized differently in every country, which might also affect a direct comparison between our study and the literature. The study by Viel et al. [17] was performed in Guadeloupe and the studies by Kuo et al. [19], Terp et al. [18] and Steel et al. [20] were performed in the United States of America, while our study was performed in the Netherlands. Although direct comparisons are difficult to make, and indirect comparisons should be interpreted with caution, these results provide us with information/indication about the safety of home telemonitoring and oxygen therapy in COVID-19 patients during a pandemic.

Besides safety from a medical perspective, it is important that patients feel safe and secure recovering at home. The large majority of the patients in our population indicated that they were satisfied recovering at home. However, we realize that only 58 of the 278 patients responded to the questionnaire. As there is in general a tendency that only the ‘extremes’ respond to a questionnaire (either the ones that are completely unsatisfied or the ones that are completely satisfied), our results are probably biased. We also would like to stress that patients receiving home telemonitoring in our study were strictly selected on inclusion criteria. Additionally, patients not feeling safe with home telemonitoring were admitted to the hospital. Therefore, our results should also be interpreted with caution and could not be extrapolated to the COVID-19 population in general.

In conclusion, we are the first in Europe to demonstrate that it appears to be safe to start home telemonitoring and oxygen therapy directly after ED assessment in COVID-19 patients. Following our results, put in perspective with literature, home telemonitoring provides the opportunity to deliver good and safe care for a subgroup of COVID-19 patients, thereby also improving hospital capacity during a pandemic. Our results encourage other hospitals to provide home telemonitoring for a selected subgroup of COVID-19 patients directly after ED presentation. Moreover, our results evoke larger evaluation studies in COVID-19 patients, also for other diseases or clinical conditions that could benefit from home telemonitoring. More knowledge about the safety and effectiveness of home telemonitoring in several specific patient groups could endorse the performance of home telemonitoring, especially during a pandemic or flu season when hospital capacity is limited.

## Figures and Tables

**Table 1:** Patient and disease characteristics of patients receiving home telemonitoring after ED assessment (ED group) and patients receiving home telemonitoring after hospital admission (admission group) at start of home telemonitoring.

	ED-Group (*n* = 65)	Admission-Group (*n* = 213)
Age (years), mean (SD)	57.1 (12.4)	59.9 (11.4)
Female, *n* (%)	28 (43.1)	82 (38.5)
BMI (kg/m^2^), mean (SD)	29.8 (5.8)	30.7 (6.9)
Normal BMI (18.5–25), *n* (%)	6 (23.1)	11 (13.4)
Overweight (25–30), *n* (%)	10 (38.5)	36 (43.9)
Obesity (≥30), *n* (%)	10 (38.5)	35 (42.7)
**Comorbidities, *n* (%)**		
Asthma	4 (6.2)	15 (7.0)
(Auto-)immune disorder	0 (0.0)	2 (0.9)
Chronic cardiovascular disease	26 (40.0)	69 (32.4)
Chronic kidney disease	1 (1.5)	7 (3.3)
Chronic liver disease	1 (1.5)	6 (2.8)
Chronic neurological disorder	7 (10.8)	19 (8.9)
Chronic pulmonary disease	0 (0.0)	12 (5.6)
Diabetes mellitus	7 (10.8)	18 (8.5)
Malignancy	12 (18.5)	32 (15.0)
COPE score death within 28 days (%) *, median (IQR) (min-max)	5.4 (2.7–9.8) (0.8–21.4) (*n* = 61)	5.3 (3.4–10.0) (0.9–24.5) (*n* = 87)
COPE score ICU admission within 28 days (%) *, median (IQR) (min-max)	14.2 (9.8–19.6) (4.9–29.6) (*n* = 61)	14.0 (11.0–19.8) (5.2–31.8) (*n* = 87)
**Medical therapy (during admission/at discharge), *n* (%)**		
Antibiotics	21 (32.3)	168 (78.9)
Anticoagulants	21 (32.3)	198 (93.0)
Steroids	24 (36.9)	197 (92.5)
Immunosuppressive drugs	1 (1.5)	8 (3.8)
Tocilizumab	3 (4.6)	34 (16.0)
Regen-cov	-	3 (1.4)
Remdesivir	-	0 (0.0)
Length of stay in days, median (IQR) (min-max)	-	4 (2–7) (0–27) **
Admission to intensive care, *n* (%)	-	12 (5.6)
**Oxygen therapy during hospitalization, *n* (%)**		
Non-invasive mechanical ventilation of high nasal flow oxygen therapy	0 (0.0)	13 (6.1)
Intubation	4 (6.2)	15 (7.0)

Abbreviation: COPE score: COVID Outcome Prediction in the Emergency Department score. * At ED assessment. ** This is an underrepresentation of the actual duration, as *n* = 5 patients were transferred to another hospital and data on those patients are not available. Therefore, admission now ended at the moment of transfer.

**Table 2:** Home telemonitoring.

	ED-Group (*n* = 65)	Admission-Group (*n* = 213)
Duration of home telemonitoring in days	14 (9–18) (2–52) ^¥^	14 (10–20) (1–91) ^¥^
**Number of measurements**		
Respiratory rate	24 (10–43) (1–131)	23 (14–42) (1–207)
O_2_ saturation	27 (14–45) (2–134)	27 (16–45) (1–204)
**Number of alerts**		
Respiratory rate	2 (0–10) (0–28)	1 (0–8) (0–95)
O_2_ saturation	3 (1–6) (0–36)	1 (0–4) (0–40)
Patients appreciating to have had an additional consultation with the physician (not urgent)	4 (1–13) (1–63)	11.5 (3–24) (1–139)
Number of telephone contacts health care provider–patient	9 (7–12) (0–27)	9 (7–12) (0–38)
Oxygen flow at start (L/min)	2 (1–2) (1–4)	2 (2–3) (1–5)
Duration of oxygen therapy in days	9 (7–13) (2–52)	10 (6–16) (1–91)
Reassessments at ED, n (%)	15 (23.8)	37 (15.8)
Hospital (re)admissions after reassessment, n (%)	10 (15.9)	14 (6.5)
ICU (re)admissions after reassessment, n (%)	5 (7.7)	5 (2.4)
Length of readmission in days	6.5 (1–8) (1–27)	5 (2–8) (0–81)
All-cause mortality, n (%)	2 (3.1)	8 (3.8)

Data are, unless indicated otherwise, presented as median (IQR) (min-max). ^¥^ In *n* = 10 patients (*n* = 5 in both groups) home telemonitoring ended within 24 h due to (re)admission.

**Table 3:** Patient experience and satisfaction.

	*n* = 58	
I received sufficient information, so I knew what I could expect.	**Did not agree** **Agreed a bit** **Partly agreed** **Largely agreed** **Agreed**	**1.9** **1.9** **5.8** **19.2** **71.2**
I received clear instructions on how to use the pulse oximeter.	**Did not agree** **Agreed a bit** **Partly agreed** **Largely agreed** **Agreed**	**0.0** **2.0** **3.9** **13.7** **80.4**
I received clear instructions on how to use the glucose meter.	**Did not agree** **Agreed a bit** **Partly agreed** **Largely agreed** **Agreed** **Not applicable**	**19.1** **0.0** **0.0** **9.5** **61.9** **9.5**
I received clear instructions on how to install the Luscii app on my mobile device.	**Did not agree** **Agreed a bit** **Partly agreed** **Largely agreed** **Agreed**	**3.9** **0.0** **2.0** **9.8** **84.3**
The Luscii app was easy to use.	**Did not agree** **Agreed a bit** **Partly agreed** **Largely agreed** **Agreed**	**2.0** **2.0** **0.0** **9.8** **86.3**
I received clear instructions on how to reach the healthcare providers.	**Did not agree** **Agreed a bit** **Partly agreed** **Largely agreed** **Agreed**	**2.0** **0.0** **5.9** **9.8** **82.4**
I could reach the healthcare providers when needed.	**Did not agree** **Agreed a bit** **Partly agreed** **Largely agreed** **Agreed**	**2.1** **2.1** **2.1** **10.6** **83.0**
Healthcare providers listened carefully to my concerns and physical complaints.	**Did not agree** **Agreed a bit** **Partly agreed** **Largely agreed** **Agreed**	**2.0** **0.0** **4.0** **10.0** **84.0**
Healthcare providers paid attention to my caregiver.	**Did not agree** **Agreed a bit** **Partly agreed** **Largely agreed** **Agreed** **I do not know**	**7.5** **5.0** **12.5** **15.0** **52.5** **7.5**
Healthcare providers had sufficient time for me and my caregiver.	**Did not agree** **Agreed a bit** **Partly agreed** **Largely agreed** **Agreed** **I do not know**	**6.7** **0.0** **4.4** **0.0** **62.2** **8.9**
The number of contacts with healthcare providers was sufficient.	**Did not agree** **Agreed a bit** **Partly agreed** **Largely agreed** **Agreed**	**2.0** **0.0** **5.9** **13.7** **78.4**
I have confidence in the expertise of healthcare providers.	**Did not agree** **Agreed a bit** **Partly agreed** **Largely agreed** **Agreed**	**2.0** **2.0** **2.0** **9.8** **84.3**
I felt safe recovering at home.	**Did not agree** **Agreed a bit** **Partly agreed** **Largely agreed** **Agreed**	**2.0** **0.0** **4.0** **8.0** **86.0**
Retrospectively, I am satisfied that I could recover at home.	**Did not agree** **Agreed a bit** **Partly agreed** **Largely agreed** **Agreed**	**0.0** **2.0** **0.0** **4.0** **94.0**
Score (0–10), median (IQR)	**9 (8–10)**	
